# Maragliano's Serum in Phthisis

**Published:** 1907-12-14

**Authors:** 


### Points in Treatment.

### MARAGLIANO'S SERUM IN PHTHISIS.

There are some physicians who have thought that Maragliano's serum did a little good in some of their cases, but the majority have given an opinion that its service is very doubtful. We propose to give a short summary of the matter, in case any of our readers should be contemplating the use of this serum in patients under their care.

Tuberculin is a vaccine and not a serum. There is not, perhaps, a universally clear understanding upon the differences between a vaccine and a serum. Briefly, they differ as follows:

A serum is obtained from the blood of an immunised animal, and to be effective it must contain, ready formed, those substances which are capable of either neutralising the poisons produced by an organism, or causing the death of the organism which produces the poisons. Those sera which contain the antidote to the toxin of the microbes—that is to say the anti-toxin—are called *anti-toxic sera*, of which anti-diphtheritic serum is a well-known example. Those sera which contain substances which kill the microbes themselves are called *anti-microbic sera*, of which Sclavo's anti-anthrax serum is an instance. A serum may be anti-toxic without being anti-microbic—that it to say, it may have the power to combine with and neutralise the toxins or poisons produced by a given kind of bacterium, although it may be unable to prevent that bacterium from continuing to grow in the patient. On the other hand, the serum may be anti-microbic without being anti-toxic—that is to say, able to kill the bacteria, but unable to neutralise the poisons already formed by it. Theoretically, a serum might be both anti-toxic and anti-microbic. The main point about a therapeutic serum, however, is that it contains the anti-body, whether anti-toxic or anti-microbic, ready formed.

A vaccine, on the other hand, itself contains no antidote either to toxin or to microbe; it contains substances which have the power to cause the tissues of the patient to produce anti-bodies in increased quantities—a very different mode of action to that of curative sera.

A serum, in other words, if it cures at all does so by a direct action on toxin or microbe, the patient being comparatively passive. A vaccine acts on the tissues of the patient, enabling them to cure themselves; the immunisation requires an active, and not a passive rôle on the patient's part.

Maragliano's serum is obtained by inoculating non-tuberculous cows with increasing quantities of tubercle bacillus cultures until they can stand what would otherwise be fatal doses. The serum is then obtained from the blood of these cows, and injected into patients as other sera are. We give a summary of the process because our readers will probably have other sera brought before them in the future, and they will wish to know how they differ from one another.

Cows were selected for the production of the serum because they are less liable to degeneration of the kidneys and to pneumonia, both of which are liable to occur in horses used for the purpose.

Each cow is first tested with tuberculin to be sure that no tuberculous lesion is present in it. Immunisation is then carried out by injections of two preparations, thought by Maragliano to contain the sum-total of the poisons formed by tubercle bacilli. These preparations are called Liquid F. and Bacillary Pulp.

Liquid F. consists of equal parts of watery tuberculin and the filtrate from bouillon cultures of tubercle bacillus fully grown—six to eight weeks old. Bacillary Pulp is made from tubercle bacilli grown on glycerine bouillon six to eight weeks old. The cultures are filtered, the filtrate being called "filtered toxine," used in Liquid F., and the bacilli are employed to make Bacillary Pulp. For this purpose they are ground in a mortar with cleaned dry sand. When sufficiently ground, cold distilled water is added, and the whole is put on to a Pasteur-Chamberland filter. The filtrate is sterile, and is supposed to contain all the filtrable substances formed in the cell of the tubercle bacillus. The finished product contains ten parts of water and one of bacilli, by weight.

Injections of these two preparations are given under the cow's skin in increasing doses, beginning with 5 c.c. We need not go into detail as to how the dosage is controlled. The cows are bled from time to time, and when the serum is found to contain 1,000 anti-toxic units per cubic centimetre it is deemed ready for therapeutic use.

Both human and bovine tubercle bacilli have been used at different times for the inoculations.

Maragliano claims that the serum acts as well given by the mouth as when it is given hypodermically. The amounts given at the Phipps Institute varied from 1 c.c. to 3 c.c. to a dose. The usual dose hypodermically was 1 c.c., and by the mouth the average was 2 c.c. Two doses per week was the rule, and in some cases the treatment was continued for as long as six months.

Over forty cases were treated at the Phipps Institute with Maragliano's serum. Most of these were in-patients, whose phthisis was extensive or severe. Some, however, were out-patients, and it was found that the serum could be used for out-patients quite well. It was given both in cases of uncomplicated phthisis and in some in whom there was also tuberculous laryngitis. Both the clinicians and the laryngologists, so far as their detailed reports can be assumed to reflect their views, seem to be doubtful of the value of the serum in the treatment of tuberculosis.

It seems clear, therefore, that Maragliano's serum is, in its present form at any rate, not to be advocated in phthisis. If it has a single point in its favour it is the stated fact that it may be administered beneficially by the mouth. One's impression is, however, that the gastric juice is almost certain to destroy any anti-bodies the serum may contain, so that the very fact that it is as serviceable by the mouth as it is hypodermically suggests strongly that it is of no specific use either way.

